# Domain‐Wall Driven Suppression of Thermal Conductivity in a Ferroelectric Polycrystal

**DOI:** 10.1002/advs.202506931

**Published:** 2025-07-25

**Authors:** Rachid Belrhiti‐Nejjar, Manuel Zahn, Patrice Limelette, Max Haas, Lucile Féger, Isabelle Monot‐Laffez, Nicolas Horny, Dennis Meier, Fabien Giovannelli, Jan Schultheiß, Guillaume F. Nataf

**Affiliations:** ^1^ GREMAN UMR 7347 Université de Tours CNRS INSA‐CVL Blois 41034 France; ^2^ Department of Materials Science and Engineering NTNU Norwegian University of Science and Technology Trondheim 7034 Norway; ^3^ Experimental Physics V Center for Electronic Correlations and Magnetism University of Augsburg 86159 Augsburg Germany; ^4^ German Aerospace Center (DLR) Institute of Materials Research 51147 Cologne Germany; ^5^ Institut de Thermique, Mécanique, Matériaux (UR 7548) Université de Reims Champagne‐Ardenne Reims 51100 France; ^6^ Department of Mechanical Engineering University of Canterbury Christchurch 8140 New Zealand

**Keywords:** domain walls, grain size, polycrystals, thermal conductivity, topological defects

## Abstract

A common strategy for reducing the thermal conductivity of polycrystalline systems is to increase the number of grain boundaries. Indeed, grain boundaries enhance the probability of phonon scattering events, which has been applied to control the thermal transport in a wide range of materials, including hard metals, diamond, oxides, and two‐dimensional (2D) systems such as graphene. Here, the opposite behavior in improper ferroelectric ErMnO_3_ polycrystals is reported, where the thermal conductivity decreases with increasing grain size. This unusual relationship between heat transport and microstructure is attributed to phonon scattering at ferroelectric domain walls. The domain walls are more densely packed in larger grains, leading to an inversion of the classical grain‐boundary‐dominated transport behavior. The findings open additional avenues for microstructural engineering of materials for thermoelectric and thermal management applications, enabling simultaneous control over mechanical, electronic, and thermal properties.

## Graphical abstract

In this study, phonon scattering at ferroelectric domain walls is used to engineer polycrystals with an unusual heat transport behavior: in the model system ErMnO_3_, thermal conductivity decreases as grain size increases, a trend opposite to that observed in other conventional materials. The work introduces domain walls as a new design principle for controlling macroscopic heat transport in polycrystals.

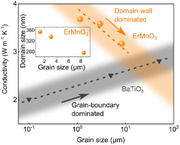

## 1 Introduction

Materials with ultra‐low thermal conductivity^[^ ^1^ ^]^ are essential for various applications, ranging from efficient thermoelectric devices^[^ ^2^, ^3^ ^]^ and thermal barrier coatings^[^ ^4^ ^]^ to insulation in cryogenic systems.^[^ ^5^ ^]^ In electrically insulating solids, strategies to reduce thermal conductivity involve hindering of phonon transport, i.e., lattice vibrations, which are the primary heat carrier.^[^ ^6^ ^]^ An established approach for decreasing the mean free path for phonons and, hence, reducing the heat flow, is to increase the number of interfaces between different phases (hetero‐interface) or grains (grain boundary)^[^ ^2^ ^]^ at which the phonons scatter. A typical example is polycrystalline thermoelectric skutterudite, where the grain boundaries reduce the thermal conductivity by about an order of magnitude compared to the single‐crystalline counterpart.^[^ ^7^ ^]^ The same trend is observed in oxides (e.g., tin oxide^[^ ^8^, ^9^ ^]^ and aluminum‐doped zinc oxide^[^ ^10^ ^]^), where the thermal conductivity can be set to values between ≈1 W m^−1^ K^−1^ and ≈40 W m^−1^ K^−1^, controlled by the density of grain boundaries. The effect extends to high thermal conductive polycrystalline materials, such as hard metals^[^ ^11^ ^]^ or diamond.^[^ ^12^ ^]^ Even in two‐dimensional (2D) materials, e.g., graphene, the thermal conductivity decreases with decreasing grain size,^[^ ^13^ ^]^ indicating that the grain‐size‐dependent scaling of thermal conductivity is largely material‐independent.

Ferroelectric and/or ferroelastic domain walls are another type of interface that strongly interacts with phonons.^[^ ^14^, ^15^, ^16^, ^17^, ^18^, ^19^, ^20^, ^21^, ^22^ ^]^ Such domain walls separate regions with different polarization and/or strain orientation. Domain wall–phonon interactions become particularly strong when the distance between the walls approaches the mean free path of the phonons,^[^ ^14^, ^15^, ^16^ ^]^ which is around 10 nm to 100 nm at room temperature in ferroelectric or ferroelastic oxides.^[^ ^14^, ^17^ ^]^ For this reason, related work mainly focuses on nanoscale domains in films with a thickness in the sub‐micrometer range.^[^ ^15^, ^16^ ^]^ One intriguing example of a bulk material where a correlation between the domain wall density and thermal transport behavior was reported is improper ferroelectric ErMnO_3_.^[^ ^23^ ^]^ The thermal conductivity of single crystals was observed to decrease along with the size of the domains, indicating the importance of the domain walls for heat transport. This observation motivates our work on ErMnO_3_ polycrystals, enabling a systematic study of the concerted impact of coexisting ferroelectric domain walls and grain boundaries.

Here, we synthesize ErMnO_3_ polycrystals with varying grain and ferroelectric domain sizes to quantify the different contributions from grain boundaries and ferroelectric domain walls on the thermal conductivity. We observe a pronounced decrease in thermal conductivity as the domain size decreases, indicating that domain walls efficiently suppress thermal conductivity. Importantly, we find that domain walls overrule grain boundary effects in ErMnO_3_, which leads to an inversion of the well‐established grain‐size‐dependent behaviour of thermal conductivity in polycrystals. Our findings show how domain wall engineering can be leveraged to control the thermal conductivity in a polycrystalline ferroelectric material, giving additional opportunities for achieving systems with ultra‐low thermal conductivity.

## 2 Tailoring Thermal Conductivity Through Domain Engineering

We begin our analysis by determining the basic crystallographic and microstructural properties of our ErMnO_3_ polycrystals, which are essential for their thermal transport behavior. X‐ray diffraction (XRD) confirms that all polycrystals have the same space group symmetry as ErMnO_3_ single crystals (*P*6_3_ *cm*,^[^ ^24^ ^]^ Figure S1a, Supporting Information), independent of the applied cooling rate. The relative geometrical densities of the samples are between 89% and 92% (**Table** **1**) as confirmed by scanning electron microscopy (SEM) images (Figure S4, Supporting Information). SEM images reveal microcrack formation caused by thermal expansion anisotropy. We note that the crack formation does not correlate with the cooling rate, as cracks form below *T*_C_,^[^ ^25^ ^]^ where all samples were cooled at the same rate. Furthermore, due to the identical cooling conditions below *T*_C_, we can exclude pronounced sample‐to‐sample variations in oxygen stoichiometry, which is predominantly controlled by dwelling times at around 350 °C.^[^ ^26^ ^]^ Grain sizes, obtained by averaging over 50−75 grains (Table 1; Figure S2 and Section S4, Supporting Information), are in the range of 9.9±0.1 µm (0.01 °C min^−1^) to 8.3±0.1 µm (10 °C min^−1^) (Table 1). Crucially, while the grain size decreases in total by ≈16% with increasing cooling rate (comparing samples cooled at 0.01 and 10 °C min^−1^), samples cooled at 0.1, 1, and 10 °C min^−1^ show nearly identical grain sizes, differing by less than 5%.

**Table 1:** Relative geometrical density, grain size, domain size, thermal diffusivity, and thermal conductivity with their standard deviations at −53 and +27 °C (room temperature). Relative geometrical densities are calculated using 7.286 g cm^−3^ as the theoretical density.^[^ ^40^ ^]^

Heat treatment conditions	Relative density (%)	Grain size (µm)	Domain size (nm)	Diffusivity (mm^2^ s^−1^)		Conductivity (W m^−1^ K^−1^)	
				−53 °C	27 °C	−53 °C	27 °C
*Cooling rate variation: Domain size decreases, grain size fixed*							
0.01 °C min^−1^	92±2	9.9±0.1	715±4	1.40±0.01	1.15±0.02	3.11±0.03	2.98±0.05
0.1 °C min^−1^	90±2	8.8±0.1	460±2	1.30±0.03	1.06±0.02	2.82±0.07	2.69±0.05
1 °C min^−1^	89±2	8.5±0.2	419±2	1.19	0.98	2.56	2.47
10 °C min^−1^	91±2	8.3±0.1	386±2	1.03±0.02	0.84±0.01	2.26±0.05	2.15±0.03
*Grain size variation: Grain size increases, domain size decreases*							
1350 °C, 10 min	85±2	1.2±0.1	351±7	1.96±0.03	1.52±0.01	4.01±0.06	3.63±0.03
1350 °C, 4 h	89±2	3.0±0.1	333±3	1.79±0.05	1.41±0.04	3.85±0.11	3.53±0.10
1450 °C, 12 h	92±2	8.4±0.2	274±2	1.53±0.06	1.22±0.04	3.39±0.13	3.15±0.09

**Figure 1:**
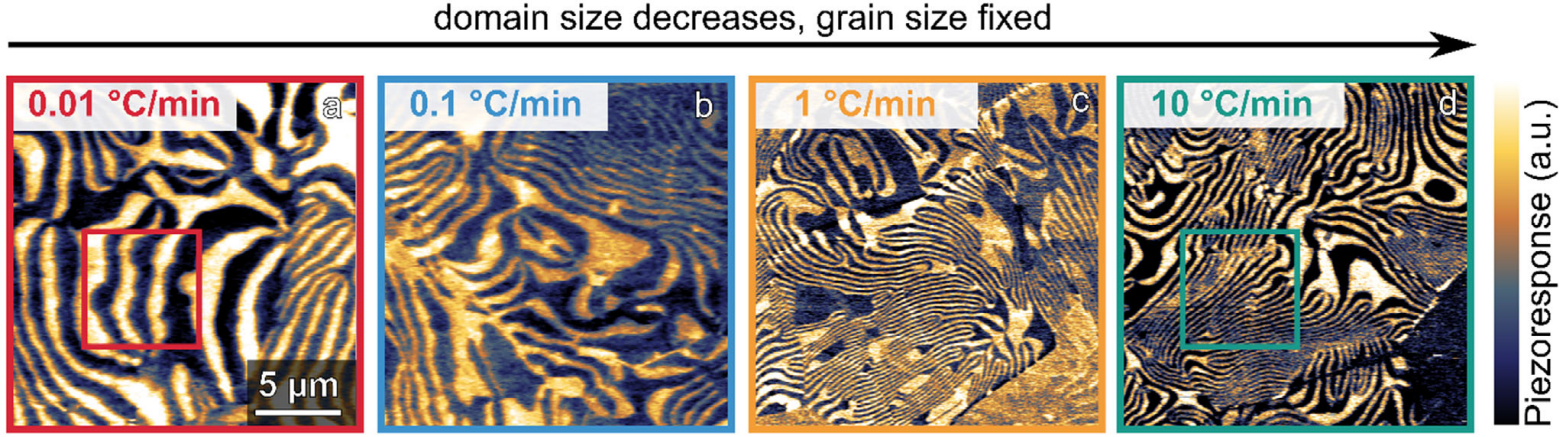
Influence of cooling rate variation on ferroelectric domain formation. PFM images of samples cooled under a) 0.01 °C min^−1^, b) 0.1 °C min^−1^, c) 1 °C min^−1,^ and d) 10 °C min^−1^ in the temperature window between 1176 °C and 1136 °C in the vicinity of *T*_C_. Data was recorded with a peak‐to‐peak excitation voltage of 10 V at a frequency of 40.13 kHz. Light and dark regions correspond to ferroelectric domains with opposite directions of polarization ±*P*. Topography data obtained on the same areas are displayed in Figure S2 (Supporting Information). A magnified view of the domain structures highlighted by the squares in panels a) and d) are shown in Figure 2b.

**Figure** **1** shows representative piezoresponse force microscopy (PFM) images of our polycrystalline ErMnO_3_ samples. ErMnO_3_ is a geometrically driven improper ferroelectric, where ferroelectric polarization, *P*, arises from a structural trimerization mode.^[^ ^27^, ^28^, ^29^ ^]^ As a uniaxial ferroelectric with *P* parallel to the hexagonal *c*‐axis (*P* ≈ 6 µC cm^−2^), its domain structure consists of 180° ferroelectric domains that converge at topologically protected six‐fold structural vortex lines as described elsewhere.^[^ ^30^ ^]^ We observe a pronounced PFM contrast that allows for distinguishing the respective +*P* and ‐*P* domains,^[^ ^31^ ^]^ showing the characteristic domain structure of polycrystalline hexagonal ErMnO_3_.^[^ ^24^ ^]^ The domain walls are characterized by atomic‐level displacements of the Er ions, separating domains of opposite ferroelectric orientation with a domain‐wall width of 6 to 8 Å.^[^ ^32^, ^33^ ^]^ A three‐dimensional (3D) characterization of the domain structure in both polycrystalline and single‐crystalline ErMnO_3_ is presented in refs. [24, 34] respectively, confirming that the characteristic domain pattern extends throughout the bulk of the material. Notably, we observe a significant cooling‐rate dependence, with the domain size decreasing as the cooling rate increases. This dependence aligns with the well‐established scaling behavior of topological defects as a function of cooling rate, known as Kibble‐Zurek scaling.^[^ ^35^, ^36^ ^]^

**Figure 2:**
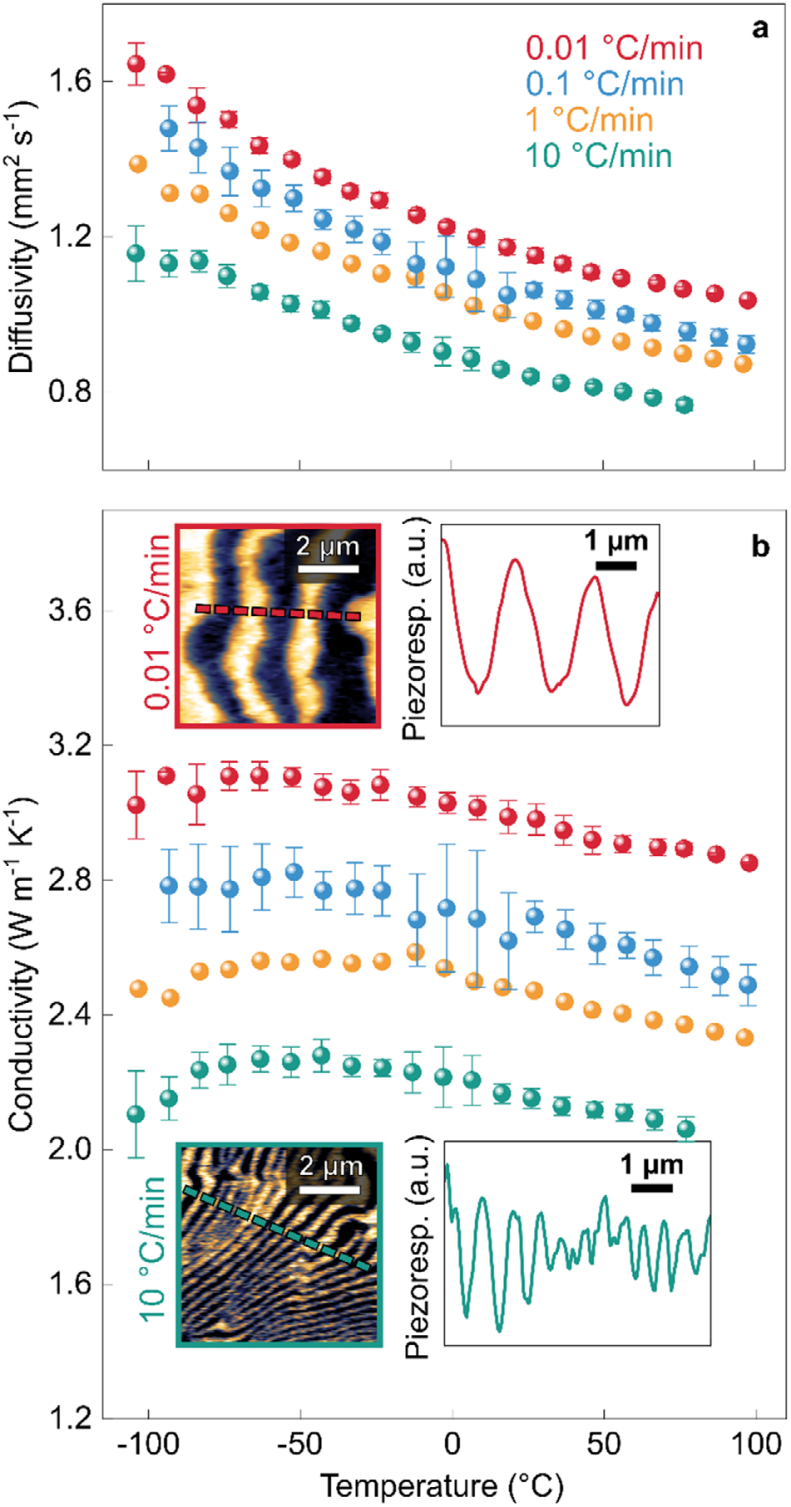
Influence of cooling rate variation on thermal conductivity. a) Thermal diffusivity and b) thermal conductivity as a function of temperature for samples cooled at different rates (recorded on heating). At each temperature, three measurements were taken, with standard deviations represented as error bars. For the sample cooled at 1 °C min^−1^, only a single measurement was performed. Carbon coating was not required, as the samples inherently absorbed light from the 650 nm laser, eliminating potential errors from variations in coating thickness. Thermal diffusivity was extracted by fitting the laser flash data to the Cape‐Lehman model.^[^ ^39^ ^]^ Across the entire temperature range, the thermal conductivity decreases by ≈25%. Representative PFM images illustrate the ferroelectric domain structure of the samples cooled at the fastest (10 °C min^−1^) and slowest (0.01 °C min^−1^) rates, corresponding to the areas highlighted in Figure 1a,d, respectively. Line plots extracted along the dashed lines visualize the dependence of domain periodicity on the cooling rate.

Thermal diffusivity is measured on the polycrystals via the laser flash method (LFA 457, Netzsch, Germany) in a temperature range from −105 to +96 °C (**Figure** **2**a). The four samples exhibit a decrease of thermal diffusivity with increasing temperature, with sample‐dependent absolute values. Subsequently, the diffusivities and geometrical densities are used to compute temperature‐dependent thermal conductivities (Figure 2b), using heat‐capacity data from ref. [23]. While thermal conductivity decreases monotonically at higher temperatures, a non‐monotonic trend emerges around −50 °C due to the temperature dependence of the heat capacity.^[^ ^23^ ^]^ At all temperatures, the thermal conductivity depends on the cooling rate through the phase transition temperature during processing. Thereby, higher cooling rates lead to greater suppression of the thermal conductivity, with a ≈25% higher thermal conductivity measured for the sample cooled under the smallest (0.01 °C min^−1^) compared to the largest (10 °C min^−1^) rate. Consistent with previous findings on single crystals of ErMnO_3_,^[^ ^23^ ^]^ we attribute the suppression of thermal conductivity with cooling rate in our polycrystals to the formation of ferroelectric domains and domain walls. The correlation between ferroelectric domain structure and thermal conductivity is displayed by representative PFM images as insets in Figure 2b for the two end cases (0.01 and 10 °C min^−1^), with the distance‐dependent piezoelectric response extracted along the dashed lines. The extracted line profiles highlight the decrease in domain size with increasing cooling rate. A detailed analysis (Section S4, Supporting Information; Table 1), averaging 50–75 grains, confirms this trend, with the slowest‐cooled sample (0.01 °C min^−1^, Figure 1a) exhibiting the largest mean domain size (≈715 nm) and the fastest‐cooled sample (10 °C min^−1^, Figure 1d) the smallest (≈386 nm). We note that hexagonal ErMnO_3_ also exhibits antiferromagnetic ordering, but only for *T* < −194 °C.^[^ ^37^ ^]^ The latter is well below the measurement range in this work and, hence, excludes magnon‐related contributions to the thermal conductivity.^[^ ^38^ ^]^ Similar to the findings on single crystals of ErMnO_3_,^[^ ^23^ ^]^ the control of thermal conductivity in our series remains effective at room temperature, with domain walls reducing the thermal conductivities from 3.0 (0.01 °C min^−1^) to 2.2 W m^−1^ K^−1^ (10 °C min^−1^). The results extend previous studies toward polycrystalline samples where domain walls coexist with additional grain boundaries, which we will explore in the next step.

## 3 Domain‐Wall Driven Suppression of Thermal Conductivity

**Figure 3:**
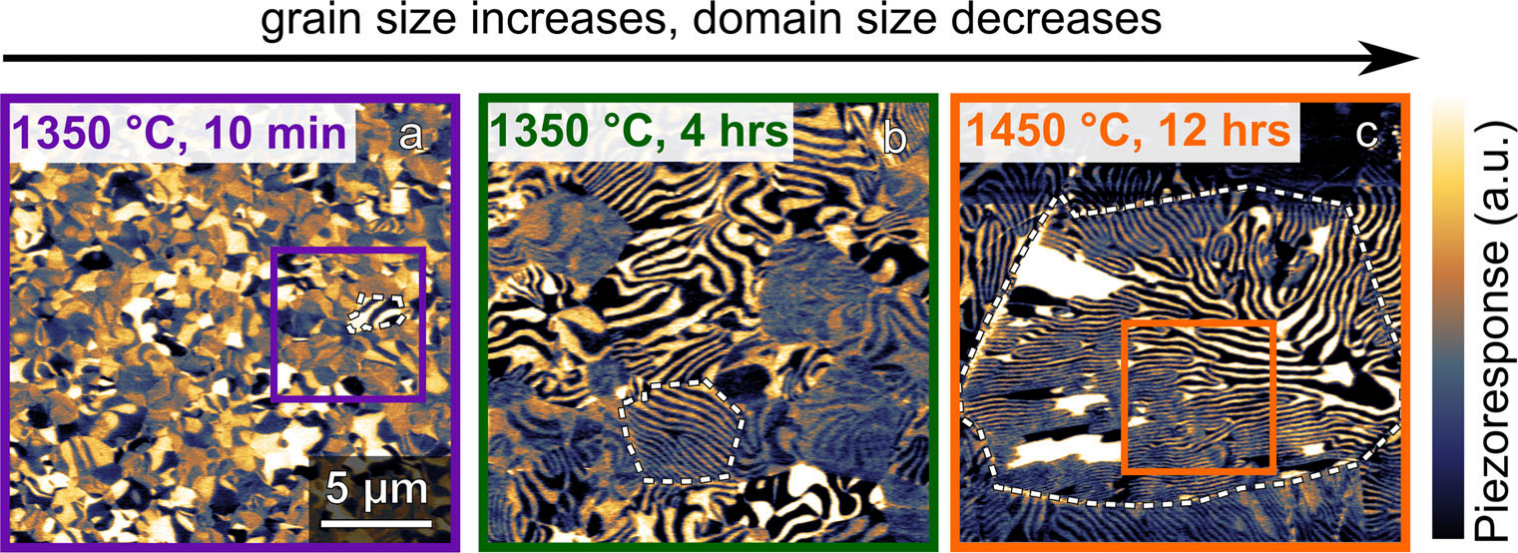
Influence of varying temperature and dwell time on ferroelectric domain formation. PFM images of samples heat treated under different temperatures and dwell times, a) 1350 °C, 10 min, b) 1350 °C, 4 h, and c) 1450 °C, 12 h. Dashed white lines indicate the position of grain boundaries. Light and dark regions correspond to ferroelectric domains with opposite directions of the polarization ±*P*. Topography data obtained on the same area is displayed in Figure S3 (Supporting Information). A magnified view of the domain structures highlighted by the squares in panels a) and d) is shown in Figure 4b.

To disentangle the contributions from domain walls and grain boundaries, we synthesize three polycrystalline samples with different grain sizes from the same powder. This is done by varying temperatures and dwell times from 1350 °C, 10 min to 1350 °C, 4 h, and 1450 °C, 12 h.^[^ ^24^ ^]^ For all samples, heating and cooling are performed at 5 °C min^−1^. XRD confirms the *P*6_3_ *cm* space group symmetry (Figure S1b, Supporting Information). The relative geometrical densities of the samples are 85% (1350 °C, 10 min), 89% (1350 °C, 4 h), and 92% (1450 °C, 12 h), as summarized in Table 1. The size of the grains is extracted from PFM data (Figure S3, Supporting Information). Averaging over 15−90 grains, we find a pronounced variation in grain size with sizes of 1.2±0.1 µm (1350 °C, 10 min), 3.0±0.1 µm (1350 °C, 4 h) to 8.4±0.2 µm (1450 °C, 12 h). **Figure** **3** shows representative PFM data, with all samples featuring a mixture of vortex and stripe‐like domains. In the sample heat treated at 1350 °C for 10 min, the formation of the vortex‐ and stripe‐like domain structure is largely suppressed (Figure 3a), i.e., the grains approach a single domain state consistent with ref. [24]. Importantly for this work, the heat treatment conditions have a significant effect on the ferroelectric domain structure, with the smallest domains observed in the sample treated at 1450 °C for 12 h. This finding is in agreement with previous observations on hexagonal manganite polycrystals,^[^ ^24^, ^41^ ^]^ where the reduction in domain size for larger grain sizes is attributed to the interaction of topological vortex lines with strain fields, facilitating the transformation into the observed stripe‐like configurations.

**Figure 4:**
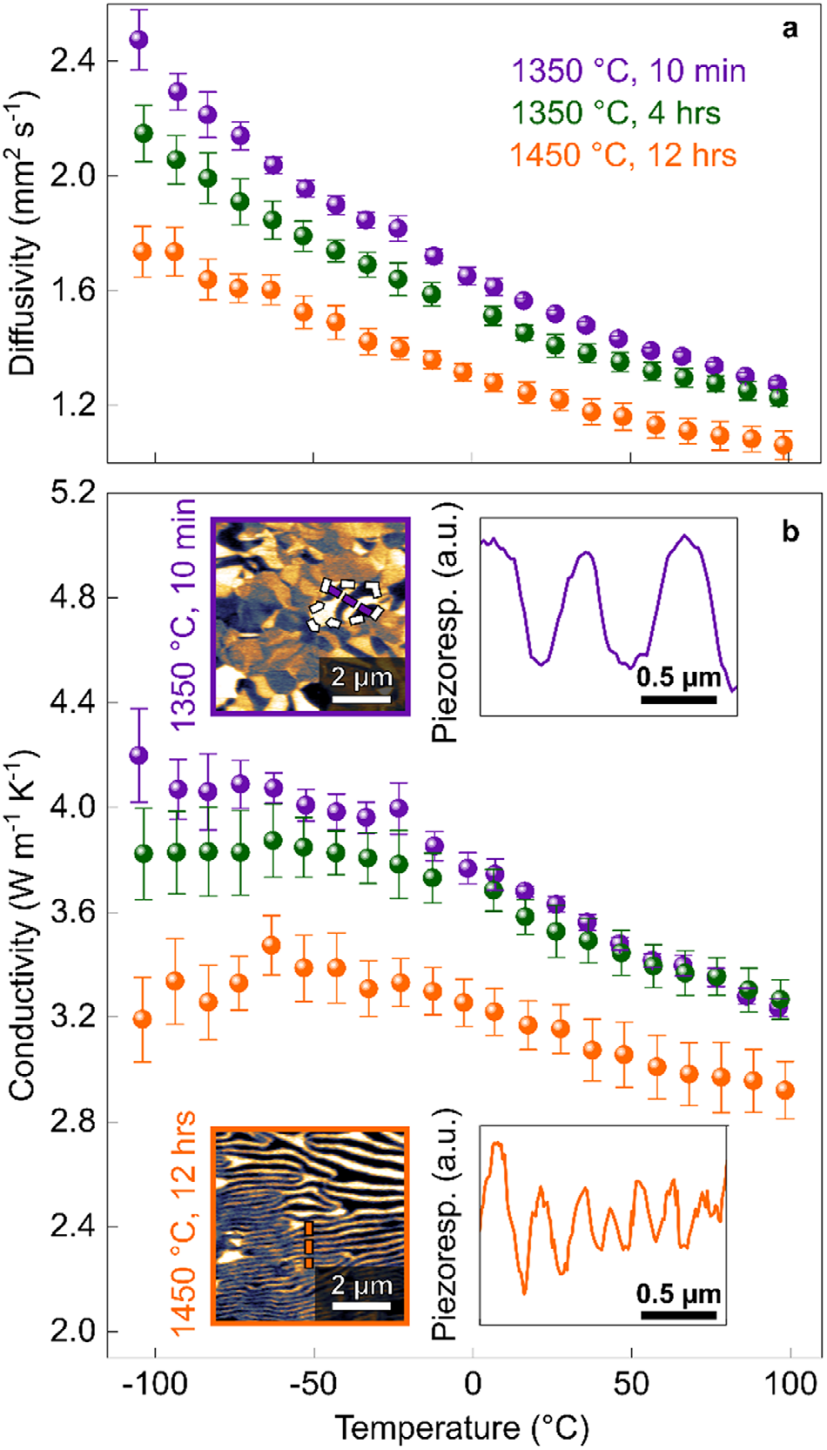
Influence of temperature and dwell time on thermal conductivity. a) Thermal diffusivity and b) thermal conductivity as a function of temperature for samples heat‐treated under different temperatures and dwell times (recorded on heating). Three measurements were taken at each temperature, with standard deviations shown as error bars. Representative PFM images show the ferroelectric domain structure of samples heat‐treated at: 1350 °C for 10 min and 1450 °C for 12 h, corresponding to the highlighted areas in Figure 3a,c, respectively. Line plots extracted along the dashed lines illustrate the relationship between domain periodicity and heat‐treatment conditions.

Thermal diffusivity (**Figure** **4**a) and thermal conductivity (Figure 4b) are measured for the three polycrystalline samples using the same procedure as described above. The samples exhibit distinct thermal conductivities, and we measure the highest value (3.6 W m^−1^ K^−1^ at room temperature) for the sample treated at 1350 °C for 10 min. In contrast, the sample treated at 1450 °C for 12 h shows a ≈12% lower thermal conductivity of 3.2 W m^−1^ K^−1^. The variation in absolute thermal conductivity values between the two sample series (Figures 2 and 4) is attributed to differences in heat treatment conditions: the samples displayed in Figures 1 and 2 (domain size decreases, grain size fixed) are sintered in a tube furnace, whereas the samples displayed in Figures 3 and 4 (grain size increases, domain size decreases) are sintered in a box furnace. Due to differences in thermal inertia between these furnaces, subtle variations in cooling rates and thermal gradients may occur, particularly at temperatures below 620 °C where oxygen content and microcrack formation are set (see Section S1, Supporting Information for details).

**Figure 5:**
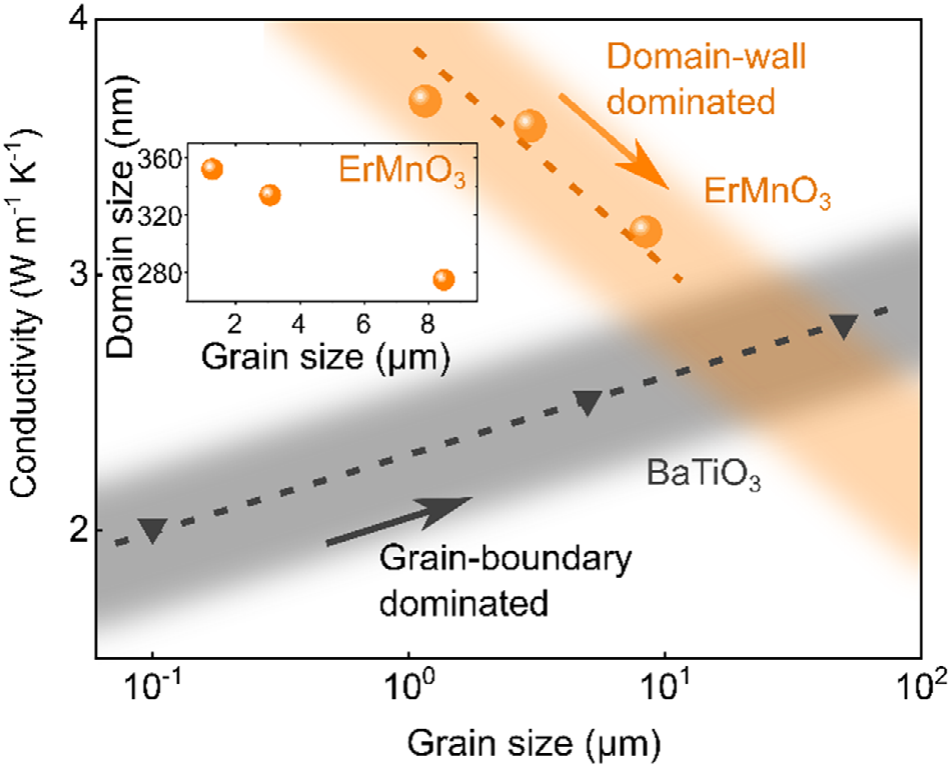
Grain‐size dependence of thermal conductivity. Grain size‐dependence for thermal conductivity at room temperature is displayed for BaTiO_3_ and ErMnO_3_. Additional temperatures and the thermal diffusivity data are plotted for ErMnO_3_ in Figure S6 (Supporting Information). For BaTiO_3_, an increase of thermal conductivity with grain size is found, indicating a common grain‐boundary‐dominated thermal conductivity. In comparison, the grain‐size‐dependence of the thermal conductivity for polycrystalline ErMnO_3_ decreases with increasing grain size, indicating the important role of domain walls for thermal conductivity control. The dashed lines indicate a guide to the eye. The inset shows the decrease in domain size with increasing grain size for ErMnO_3_, highlighting the dominant role of ferroelectric domain walls for the thermal conductivity.

The relationship between thermal conductivity and grain size is displayed in **Figure** **5**. Unlike in conventional polycrystalline materials, we find that the thermal conductivity in ErMnO_3_ decreases with increasing grain size. This inverse trend is in contrast to literature data on, e.g., polycrystalline SnO_2_,^[^ ^9^ ^]^ Al‐ZnO,^[^ ^10^ ^]^ and WC‐Co,^[^ ^11^ ^]^ where thermal conductivity increases with grain size. This unusual behavior suggests that the grain‐size dependence of thermal conductivity is governed by a mechanism other than the conventional phonon scattering at grain boundaries. Based on the results reported for single crystals,^[^ ^18^ ^]^ and our data gained on polycrystalline samples (Figures 1 and 2), we conclude that this behavior originates from the varying domain wall density (see insets and line profiles in Figure 4b). The line profiles reflect that the sample treated at the highest temperature (1450 °C for 12 h) has a smaller domain size compared to the sample treated at the lowest temperature (1350 °C for 10 min). A more detailed analysis (Table 1) confirms this trend, with mean domain sizes of 351 nm (1350 °C, 10 min), 333 nm (1350 °C, 4 h), and 274 nm (1450 °C, 12 h). For reference, our results are plotted together with grain‐size‐dependent thermal conductivity data we gained on the model ferroelectric BaTiO_3_ (grain size range of 0.1 to 50 µm). BaTiO_3_ shows the classical relation expected for polycrystals,^[^ ^9^, ^10^, ^11^, ^42^ ^]^ and the comparison between as‐grown BaTiO_3_ and ErMnO_3_ emphasizes the unusual behavior of our ErMnO_3_ polycrystals.

To quantify for the contributions, we apply a one‐dimensional (1D) heat transfer model, where the thermal resistivity of the samples is described as the sum of resistances in series, corresponding to the grain boundaries and domain walls, and the intrinsic thermal resistance of the material *R*_i_ = *e*/κ_i_ , with *e* being the thickness of the sample and κ_i_ the intrinsic thermal conductivity. The effective thermal conductivity is thus expressed as:

(1) $$\;\kappa =\frac{1}{\frac{R_{\mathrm{GB}}}{l_{\mathrm{GB}}}+\left(\frac{1}{l_{\mathrm{DW}}}-\frac{1}{l_{\mathrm{GB}}}\right)R_{\mathrm{DW}}+\frac{1}{\kappa_{i}}}\;,$$

where *l*_GB_ and *l*_DW_ are the grain and domain sizes, 1/*l*_GB_ and (1/*l*_DW_ − 1/*l*_GB_) multiplied by *e* is the number of grains and domains, respectively. *R*_GB_ and *R*_DW_ are the respective Kapitza resistances,^[^ ^43^ ^]^ measuring the resistance to thermal flow at the interface between two grains and two domains, respectively.

Assuming that the samples have a similar κ_i_, the resistances *R*_GB_ and *R*_DW_ can be estimated at each temperature by minimizing a cost function built as the sum of the squares of the difference between each κ_i_ calculated using Equation 1. Utilizing experimentally obtained values κ from thermal flash measurements (Figures 2 and 4), and *l*_GB_ and *l*_DW_ from PFM measurements (Figures 1 and 3), the temperature‐dependent thermal conductivity with a sample‐independent κ_i_ can be consistently described (Figure S7, Supporting Information). This thermal model allows for estimating thermal resistances between grains and domains, with mean values *R*_GB_ =  1.6 · 10^−9^ m^2^ K W^−1^ and *R*_DW_ =  2.8 · 10^−8^ m^2^ K W^−1^. These values align well with interfacial thermal resistances found in other oxides, such as 5 · 10^−9^ m^2^ K W^−1^ for grain boundaries in SrTiO_3_ polycrystals.^[^ ^44^ ^]^ Thermal resistances for domain walls fall within a similar range of 5 · 10^−9^ m^2^ K W^−1^ for PbTiO_3_^[^ ^16^ ^]^ and 2 · 10^−8^ m^2^ K W^−1^ for BiFeO_3_.^[^ ^22^ ^]^ Importantly, the model corroborates that both ferroelectric domain walls and grain boundaries have a substantial impact on the thermal conductivity in our ErMnO_3_ polycrystals. The difference in resistivities (*R*_DW_ *> R*_GB_) is consistent with our experimental observations and indicates that the domain walls suppress thermal conductivity more efficiently than the grain boundaries. Possible scattering centers for the phonons include polarization discontinuities at the ferroelectric domain walls as well as point defects, which segregate toward domain walls in hexagonal manganites.^[^ ^45^ ^]^ Future work may provide additional insight into the phonon scattering mechanism, e.g., by targeted manipulation of oxygen defects through annealing experiments.^[^ ^26^ ^]^

## 4 Conclusion

In contrast to classical polycrystalline materials – where grain boundaries govern the grain‐size dependence of thermal conductivity – our study demonstrates an inverted behavior in ErMnO_3_ polycrystals. Through a comprehensive analysis of samples with similar grain sizes but different domain sizes, we show that ferroelectric domain walls effectively suppress thermal conductivity by impeding phonon transport. Most interestingly, this effect is evident as an observed decrease in thermal conductivity with increasing grain size, where the influence of domain walls overrides the decreasing grain boundary scattering effects. To quantify this unusual behavior, we calculated the Kapitza resistance, which was found to be higher for the domain walls than for the grain boundaries, corroborating the dominant effect of domain walls on the thermal conductivity of ErMnO_3_ polycrystals.

The inversion of thermal conductivity scaling with grain size is not limited to ErMnO_3_ and can be extended to other polycrystalline materials with ferroelectric and/or ferroelastic domain walls, where a similar domain‐size/grain‐size scaling behavior naturally occurs in the as‐grown state or can be introduced, e.g., via strain‐engineering concepts.^[^ ^46^ ^]^ Potential candidates for the former case include isostructural hexagonal indates,^[^ ^47^ ^]^ tungsten bronzes,^[^ ^48^ ^]^ and 2H compounds,^[^ ^49^ ^]^ which share the same hexagonal *P*6_3_ *cm* crystal structure. The impact of ferroelectric domain walls on the grain‐size dependence of thermal conductivity yields an innovative approach for tuning thermal properties. Our findings introduce an additional lever for adjusting thermal conductivity beyond traditional grain size modulation, opening an avenue for designing microstructures with tailored thermal behaviors. Besides ferroelectric and/or ferroelastic domain walls, other phonon‐scattering interfaces, that can be engineered independently of grain size, such as magnetic domain walls^[^ ^50^ ^]^ or twin boundaries,^[^ ^51^, ^52^ ^]^ are expected to exhibit a similar effect. Properties such as fracture toughness, which typically improves with grain size,^[^ ^53^ ^]^ could be optimized for ultra‐low thermal conductivity applications. Gradient grain size distributions^[^ ^54^ ^]^ further allow for localized control, providing a microstructure‐related parameter that may be leveraged to generate inhomogeneous heat flow in electronics and thermoelectrics.

## Conflict of Interest

The authors declare no conflict of interest.

## Author Contributions

R.B.‐N. and M.Z. contributed equally to this work. G.F.N., F.G., J.S., and D.M. conceived the study. R.B.‐N. performed thermal conductivity measurements on ErMnO_3_ polycrystals and analyzed the data under the supervision of G.F.N. and F.G. M.H. and J.S. synthesized the ErMnO_3_ polycrystals. L.F. synthesized the BaTiO_3_ polycrystals under the supervision of I.M.‐L. and measured their thermal conductivity under the supervision of I.M.‐L. and G.F.N. P.L. and N.H. developed the Kapitza model. M.Z. developed the procedure for domain and grain size quantification, supervised by J.S. G.F.N. and J.S. wrote the manuscript together with D.M. All authors contributed to the discussion of the results and the final version of the manuscript.

## Supporting information



Supporting Information

## Data Availability

The data that support the findings of this study are available from the corresponding author upon reasonable request.
