# Supplementary material for: Domain‐Wall Driven Suppression of Thermal Conductivity in a Ferroelectric Polycrystal
Source: Adv Sci (Weinh). 2025 Jul 25;12(38):e06931. doi: 10.1002/advs.202506931 (PMC12520512; doi:10.1002/advs.202506931)
Supplement: Supplementary file 1 — Supporting Information [file ADVS-12-e06931-s001.docx]

**Supporting Information**

**Domain-wall driven suppression of thermal
conductivity in a ferroelectric polycrystal**

Rachid Belrhiti-Nejjar^1,*^, Manuel Zahn^2,3*^, Patrice Limelette^1^, Max Haas^2,4^, Lucile Féger^1^, Isabelle Monot-Laffez^1^, Nicolas Horny^5^, Dennis Meier^2^, Fabien Giovannelli^1,**^, Jan Schultheiß^2,6,***^, Guillaume F. Nataf^1,****^

^1^ GREMAN UMR 7347, Université de Tours, CNRS, INSA-CVL, 15 rue de la chocolaterie, 41034 Blois, France

^2^ Department of Materials Science and Engineering, NTNU Norwegian University of Science and Technology, Trondheim 7034, Norway

^3^ Experimental Physics V, Center for Electronic Correlations and Magnetism, University of Augsburg, 86159 Augsburg, Germany

^4^ German Aerospace Center (DLR), Institute of Materials Research, 51147 Cologne, Germany

^5^ Institut de Thermique, Mécanique, Matériaux (UR 7548), Université de Reims Champagne-Ardenne, 51100 Reims, France

^6^ Department of Mechanical Engineering, University of Canterbury, 8140 Christchurch, New Zealand

* equal contributions
** fabien.giovannelli@univ-tours.fr
*** jan.schultheiss@ntnu.no
*** guillaume.nataf@univ-tours.fr

**S1. Synthesis and characterization of polycrystalline ErMnO_3_ and BaTiO_3_**

ErMnO_3_ powder was obtained by a solid-state reaction between Er_2_O_3_ (99.9% purity; Alfa Aesar, MA, USA) and Mn_2_O_3_ (99.0% purity; Sigma-Aldrich, Germany). Powders were dried at 900 °C and 700 °C for 12 hours, mixed in a stoichiometric ratio and ball milled (BML 5, witeg Labortechnik GmbH, Germany) for 12 hours at 205 rpm using yttria stabilized zirconia milling balls with a diameter of 5 mm and ethanol as dispersion medium. After drying, the resulting powder was annealed at 1000 °C, mortared, annealed at 1050 °C, mortared again and finally annealed at 1100 °C, every time for 12 hours. To study the impact of domain walls on thermal conductivity in polycrystals of ErMnO_3_, powder was synthesized via a solid-state reaction followed by densification into bulk polycrystals as described in ref. [1].

To obtain dense materials similar in grain size, heat treatment was carried out at 1475 °C for 24 hrs, with a heating and cooling rate of 5 °C/min. Ferroelectric domain size control was achieved via variations in the cooling rates between 0.01 and 10 °C/min (0.01, 0.1, 1, and 10 °C/min) in the temperature range of 1176 °C and 1136 °C, near the ferroelectric transition, *T*_C_ = 1156 °C,^[2]^ following established procedures described in refs. [2, 3] for single crystals. For realizing the different cooling rates (Fig. 1 and 2), sintering was carried out in a tube furnace (ETF 17 horizontal tube furnace, Entech, Ängelholm, Sweden, 40 mm in diameter). Before laser flash measurements, these samples were lapped with a 9-µm-grained Al_2_O_3_ water suspension (Logitech Ltd, Glasgow, Scotland) followed by polishing with silica slurry (SF1 Polishing Fluid, Logitech AS, Glasgow, Scotland).

To obtain the series with different grain sizes (Fig. 3 and 4), sintering was carried out in a box furnace (Entech Energiteknik AB, Ängelholm, Sweden, SF 5, 165 x 160 x 270 mm) at temperatures ranging between 1350 °C and 1450 °C. Before laser flash measurements, these samples were mechanically polished. Thermal diffusivity was measured with a laser flash setup (LFA 457, Netzsch, Germany). Relative densities were obtained geometrically and by the Archimedes method (MS204TS/00 analytical balance, Mettler Toledo, Switzerland) in distilled water.

The BaTiO_3_ polycrystals were synthesized from commercial BaTiO_3_ nano- (purity 99.99%, Chempur, Poland) and micro-powders (purity 99.5%, Sigma-Aldrich, Germany), mixed with 5% polyvinyl alcohol (2 wt.% in water, VWR International, USA), and then densified by conventional sintering at 1500 °C for different dwell times to obtain mean grain sizes of 5 µm (1400 °C, 1 hrs) and 50 µm (1400 °C, 6 hrs). A polycrystal with an average grain size of 0.1 µm was obtained by spark plasma sintering using a SPS632Lx (Dr.Sinter, Fuji Electronics, Japan). Sintering was performed with a graphite mold at 1050 °C for 3 min with a heating rate of 100 °C min^-1^ under a uniaxial pressure of 100 MPa. Subsequently, the pellet was post-annealed for 1 hour under oxygen flow at 1000 °C in a tube furnace to compensate for oxygen losses due to reducing conditions in the spark plasma sintering process. Thermal diffusivity was measured with the same laser flash setup as for the ErMnO_3_ polycrystal (LFA 457, Netzsch, Germany). Silver glue paint and a thin graphite coating were used to maximize the infrared absorption and emission. The specific heat capacity was measured near room temperature on crushed polycrystals by differential scanning calorimetry (STA 449 F3 Jupiter, Netzsch, Germany) in platinum crucibles and nitrogen atmosphere. The relative density of the three polycrystals of BaTiO_3_, obtained by the Archimedes method, were: 94% (0.1 µm grains), 97% (5 µm grains) and 94% (50 µm grains) considering a theoretical density of 6.02 g cm^-3^.

**S2. Crystallographic analysis of ErMnO_3_ polycrystals**

X-Ray Diffraction (XRD, D8 ADVANCE, Bruker, MA, USA) of the investigated ErMnO_3_ polycrystals is shown in Fig. S1. The crystallographic structures and phases are observed to be independent of the heat-treatment conditions and can be described with a hexagonal structure with space group symmetry *P*6_3_*cm*, similar to an ErMnO_3_ single crystal.^[4]^ No secondary phases can be observed.


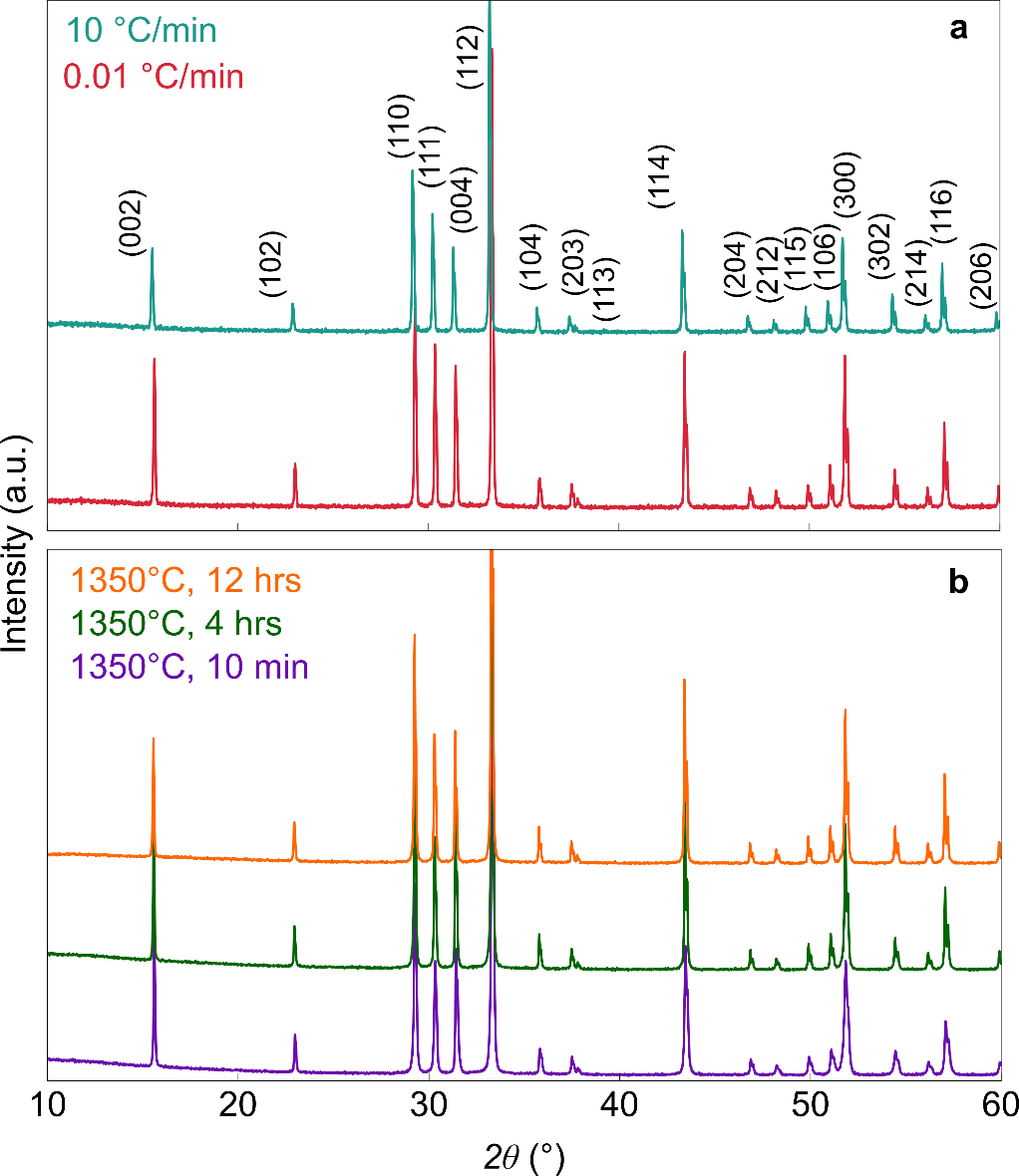


**Figure S1.** XRD patterns of polycrystalline pellets cooled under a) different rates (10 °C/min and 0.01 °C/min) and b) different heat-treatment conditions (1350 °C, 10 min; 1350 °C, 4 hrs; and 1450 °C, 12 hrs). The data demonstrates that all samples have a hexagonal structure with space group symmetry *P*6_3_*cm*.^[4]^

**S3. Analysis of micro- and domain structure**

To visualize the micro- and domain structure of the samples, lapping was done with a 9-µm-grained Al_2_O_3_ water suspension (Logitech Ltd, Glasgow, Scotland) followed by polishing with silica slurry (SF1 Polishing Fluid, Logitech AS, Glasgow, Scotland). Piezoresponse force microscopy (PFM) measurements were performed on an Ntegra Prisma system (NT-MDT, Moscow, Russia), with an electrically conductive platinum tip (Spark 150 Pt, Nu Nano Ltd, UK). The sample was excited using an alternating voltage (40.13 kHz, 10 V peak-to-peak). The laser deflection was read out by lock-in amplifiers (SR830, Stanford Research Systems, CA, USA). Topography data for the two different sample series is displayed in Fig. S2 (corresponds to PFM data displayed in Fig. 1) and Fig. S3 (corresponds to PFM data displayed in Fig. 3).


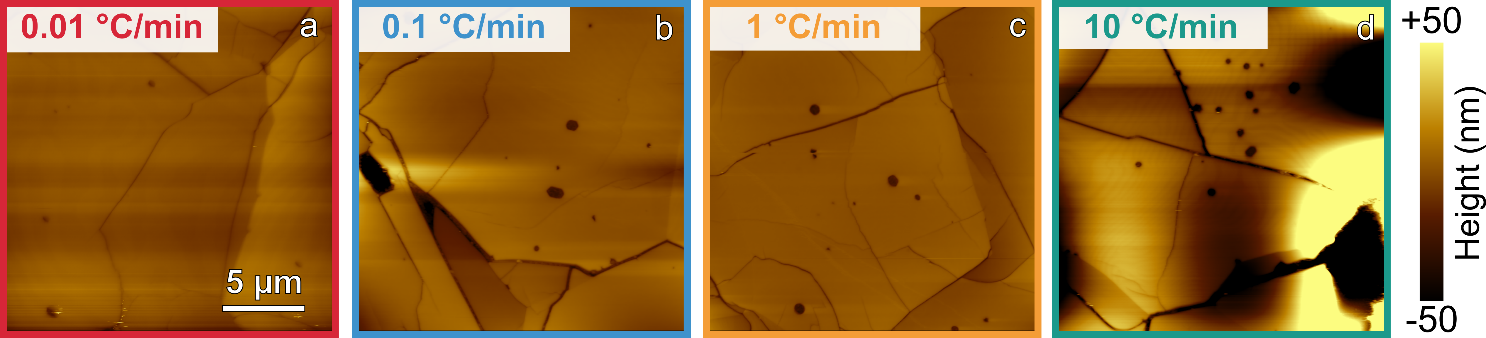


**Figure S2.** Topography data of the samples cooled at a) 0.01 °C/min, b) 0.1 °C/min, c) 1 °C/min, and d) 10 °C/min, covering the same area as shown for the PFM data in Fig. 1.


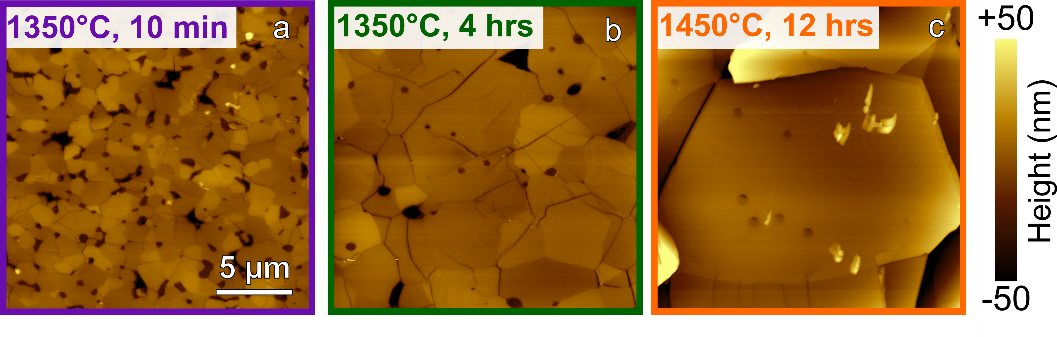


**Figure S3.** Topography data of the samples treated under different temperatures and dwell times, a) 1350 °C, 10 min, b) 1350 °C, 4 hrs, and c) 1450 °C, 12 hrs, covering the same area as shown for the PFM data in Fig. 3.

The microstructure of the polycrystals, investigated by scanning electron microscopy (Tescan Mira 3, Czech Republic) operating in secondary electrons (SE) mode with an acceleration voltage of 5 kV is displayed in Fig. S4.


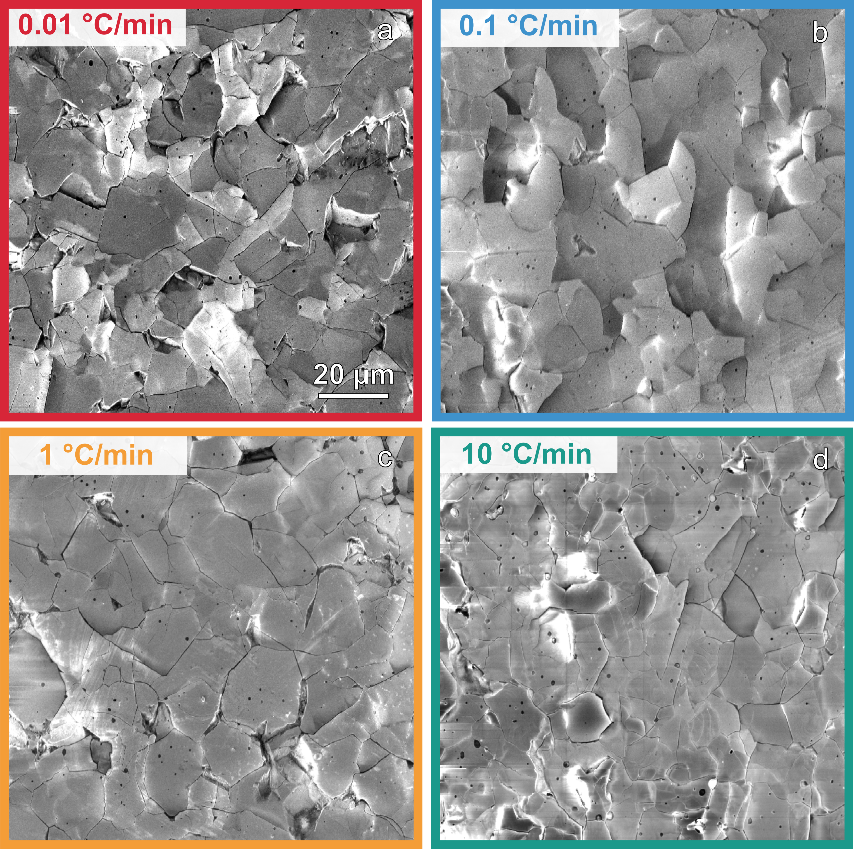


**Figure S4.** SEM micrographs of the samples cooled at a) 0.01 °C/min, b) 0.1 °C/min, c) 1 °C/min, and d) 10 °C/min.

**S4. Grain size and domain size determination**

To obtain the domain size distribution, a stereographical method^[5]^ is applied. The overall procedure, starting from the raw PFM data, is visualized for an exemplary grain in Fig. S5. First, for each grain, the shape of the grain boundary is manually determined based on the PFM image and the simultaneously recorded topography data (dashed line in Fig. S5a). Using the mean PFM signal within the entire grain as the threshold, a binarized image is calculated as shown in Fig. S5b.

Next, the domain walls are determined as the edges within the binarized image using a standard Canny edge algorithm^[6]^ displayed in Fig. S5c. Subsequently, the distance of each pixel within the grain to the nearest pixel belonging to a domain wall (Fig. S5c) *d*_p-dw_ is determined, leading after spatial averaging to an estimated mean domain size *d*_estimate_ at each position as shown in Fig. S5d. The spatial averaging ensures that pixels close to a domain wall also recognize the distance to the next nearest domain wall and is calculated for each pixel *p* as

| $d_{\mathrm{estimate}}(p)=\max_{p^{'} \in\mathrm{grain}}\left\{ \begin{matrix} d_{p-dw}\left( p' \right), & \mathrm{if}\left\vert p-p^{'} \right\vert<3d_{p-dw}(p') \\ 0, & \mathrm{else} \end{matrix} \right.$. | (S1) |
| --- | --- |

Based on the initial estimated domain size, the local mean value of the PFM signal within a circle with radius three times the estimated domain size is calculated for each pixel and applied as the threshold for local binarization in the second iteration, generating a new version of Fig. S5b. Following the previously described steps through the cycle leads to an updated local domain size (Fig. S5d) that is compared to the local domain size from the previous iteration. If the average estimated domain size changes by more than 1 ‰ within an iteration, another iteration is performed. After several iterative cycles, the estimated domain size saturates due to the achieved consistency between binarization and domain size estimation. This leads to the final representation of the domain walls shown in Fig S5e. The iterative sequence is required to account for local changes of the PFM background signal, e.g. due to topographic crosstalk, that cannot be captured by a global threshold value. Our algorithm builds up on existing approaches^[7, 8]^ for variable-scale local thresholding developed in the field of image and document recognition and extends them towards the application of domain size determination in ferroelectrics.

After optimization, as shown in the inset of Fig. S5e intersections of straight lines with the domain walls (spacing of 470 nm between lines, repeated for 20 grids, each one rotated by 9° with respect to the previous one) and their respective distance are determined. This leads to a distribution of the lengths between the intersections, which we use for domain size quantification, as visualized in a histogram in Fig. S5f. Using Maximum-Likelihood optimization^[9]^, the Gamma distribution *g*(*d*), best fitting the measured distances *d* given by

| $g_{b,p}(d)=\frac{b^{p}}{\Gamma(p)}\cdot d^{(p-1)}\cdot\exp(-b\cdot d)$ | (S2) |
| --- | --- |

with the rate parameters *b* and the shape parameter *p* is determined. The probability distribution is chosen due to their application in Queueing theory on similar problems of sequentially occurring events with defined average probability of occurrence^[10]^. Finally, the distance expectation value

| $\overline{d}=\int_{0}^{\infty} d'\cdot g_{b,p}(d')\text{d}d'=p/b$ | (S3) |
| --- | --- |

and the respective uncertainty (due to the uncertainty of *b* and *p*) are used as the measure of the average domain size.

To calculate the mean grain size, the last part of the algorithm (after the last iterative cycle) is applied to the manually drawn outer shape of the grain instead of the final domain wall representation (Fig. S5e). Thereby the intersection points of the lines with the grain boundary can be directly calculated followed by the probability distribution-based calculation of the mean size.


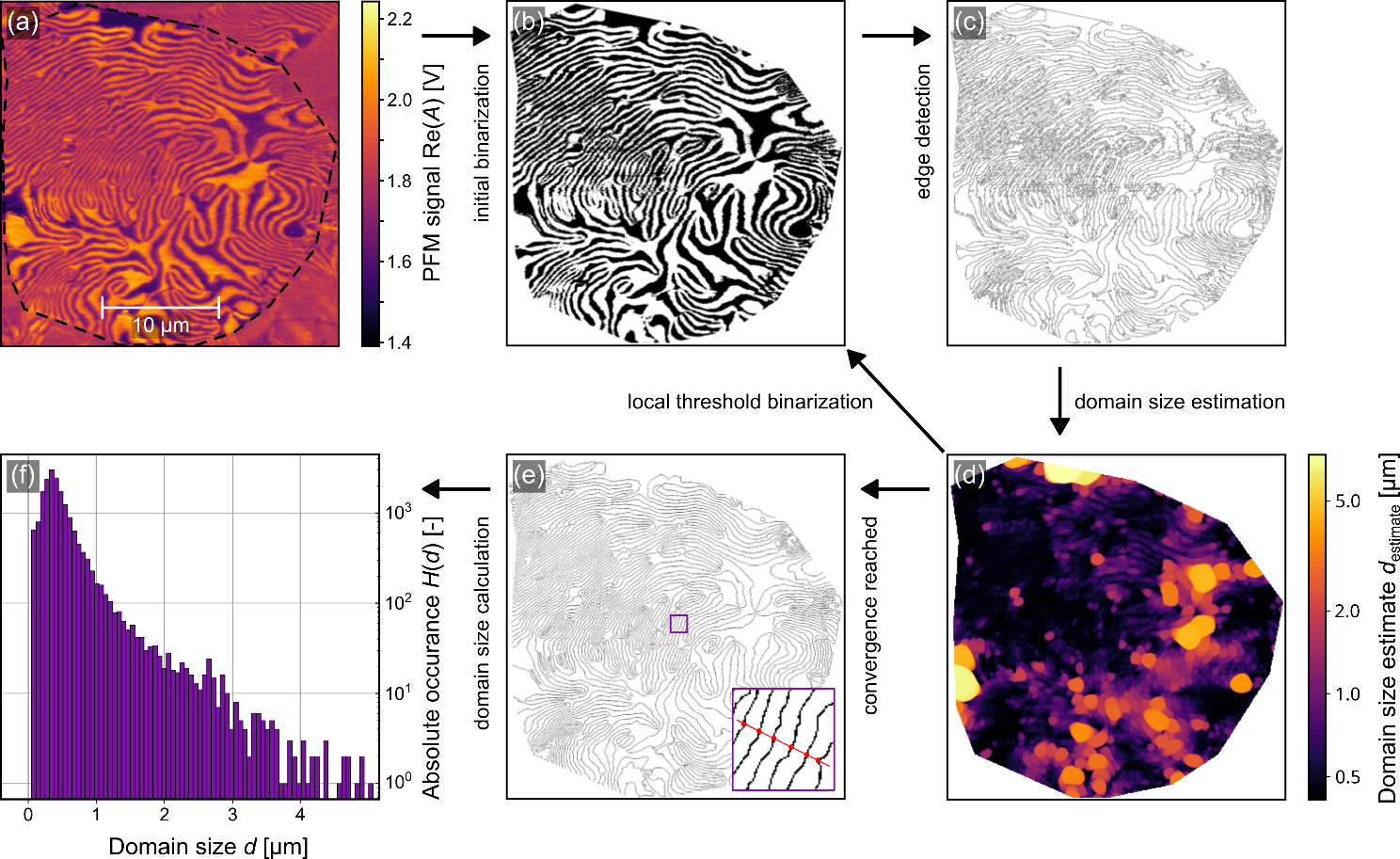


**Figure S5.** Steps of image processing algorithm for domain size determination. a) PFM image of a typical domain pattern with the dashed black line visualizing the grain boundary, b) binarized image, c) extracted domain walls, d) estimated local domain size, e) final extracted domain walls and f) final domain size distribution for the PFM image in a). The inset in e) shows the intersection of a straight line with the domain walls as applied in the final domain distance calculation step.

**S5. Grain size dependence of the thermal diffusivity and thermal conductivity**

A visualization of the thermal diffusivity and thermal conductivity as a function of grain size for different temperatures is displayed in Fig. S6.


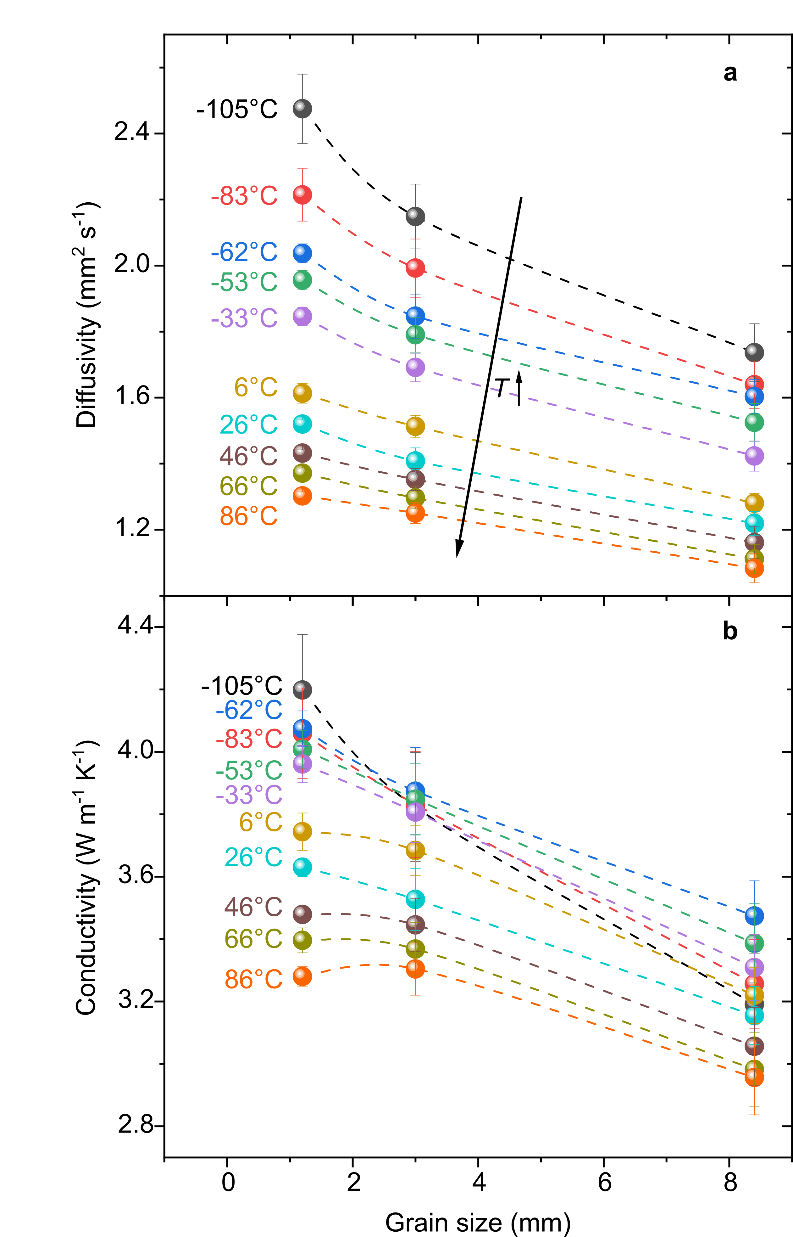


**Fig. S6. Conductivity and diffusivity as a function of grain size measured at different temperatures.** Particularly at lower temperatures, where phonon scattering at domain walls is more effective, a clear decrease of a) thermal diffusivity and b) thermal conductivity with grain size can be observed.

**S6. One-dimensional heat transfer model**

Assuming a constant intrinsic thermal conductivity $\kappa_{i}$ for all samples, the thermal boundary resistances $R_{\mathrm{GB}}$ and $R_{\mathrm{DW}}$ are estimated for each temperature, following Eq. 1 (Fig. S7a). The obtained mean values are $R_{\mathrm{GB}}=1.6\times{10}^{-9}$ m^2^ K W^-1^ and $R_{\mathrm{DW}}=2.8\times{10}^{-8}$ m^2^ K W^-1^. The corresponding intrinsic thermal conductivities $\kappa_{i}$ for each sample and temperature are shown in Fig. S7b. The variation in absolute values between the two samples series is attributed to differences in heat treatment conditions (see Supplementary Section S1 for more details). For each series, the standard deviation is in the range from 3 to 10%, confirming similar intrinsic thermal conductivity among the samples.


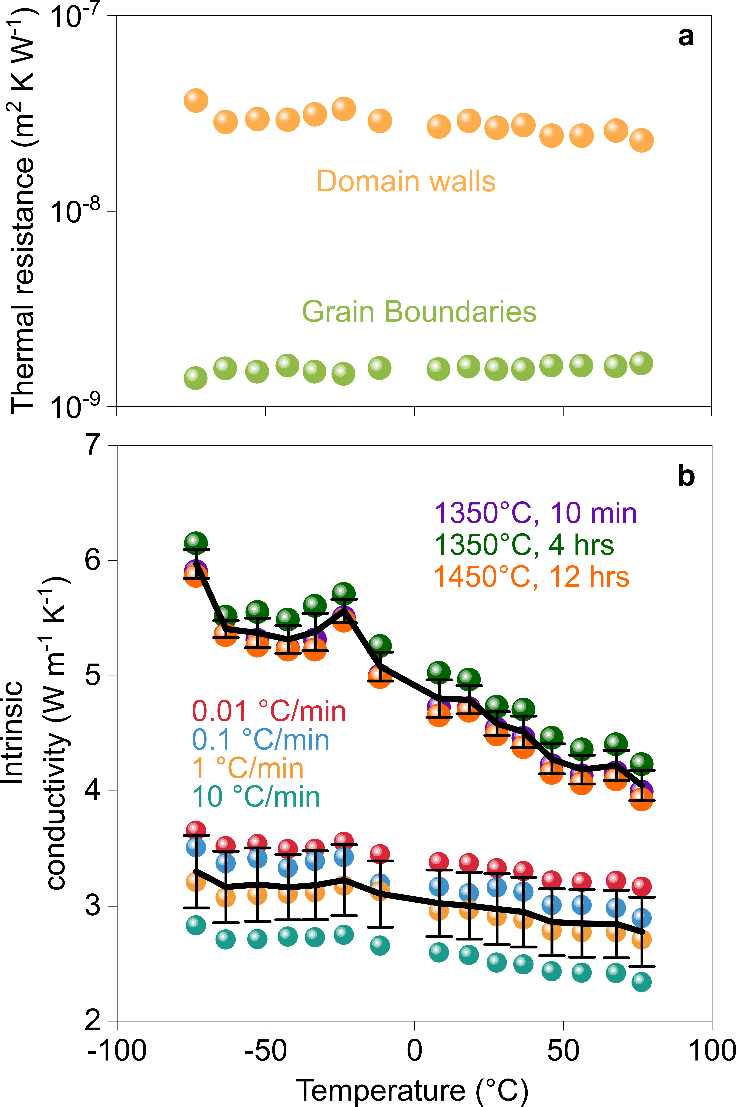


**Figure S7.** a) Estimated temperature-dependent thermal resistances, $R_{\mathrm{DW}}$ and $R_{\mathrm{DW}}$, for domain walls and grain boundaries, respectively. b) Temperature-dependent intrinsic thermal conductivity is calculated according to Eq. (1) utilizing experimentally determined $\kappa$ values, grain and domain sizes from PFM data, and estimated thermal resistances. Black lines indicate the average value for each series, with standard-deviation displayed as error bars.

**References**

[1] J. Schultheiß, F. Xue, E. Roede, H. W. Ånes, F. H. Danmo, S. M. Selbach, L.-Q. Chen, D. Meier, *Adv. Mater.* **2022**, *34*, 2203449.

[2] S. C. Chae, N. Lee, Y. Horibe, M. Tanimura, S. Mori, B. Gao, S. Carr, S. W. Cheong, *Phys. Rev. Lett.* **2012**, *108*, 167603.

[3] S. M. Griffin, M. Lilienblum, K. T. Delaney, Y. Kumagai, M. Fiebig, N. A. Spaldin, *Phys. Rev. X* **2012**, *2*, 041022.

[4] B. B. Van Aken, A. Meetsma, T. T. Palstra, *Acta Crystallogr., Sect. E: Struct. Rep. Online* **2001**, *57*, i38.

[5] A. Hubert, R. Schäfer, *Magnetic Domains: The Analysis of Magnetic Microstructures*, Springer, Berlin, Heidelberg, **2008**.

[6] J. Canny, *IEEE Trans. Pattern Anal. Mach. Intell.* **1986**, 679.

[7] C.-A. Boiangiu, A. Olteanu, A. Stefanescu, D. Rosner, N. Tapus, M. I. Andreica, "Local thresholding algorithm based on variable window size statistics", presented at *Proceedings of the 18th International Conference on Control Systems and Computer Science*, **2011**.

[8] L. P. Saxena, *Artificial Intelligence Review* **2019**, *51*, 673.

[9] L. Held, D. Sabanés Bové, *Likelihood and Bayesian Inference*, Springer, Berlin, Heidelberg, **2014**.

[10] D. Gross, J. F. Shortle, J. M. Thompson, C. M. Harris, *Fundamentals of Queueing Theory*, John Wiley & Sons, Hoboken, NJ, **2011**.
